# A short-chain fatty acid, propionate, enhances the cytotoxic effect of cisplatin by modulating GPR41 signaling pathways in HepG2 cells

**DOI:** 10.18632/oncotarget.25809

**Published:** 2018-07-31

**Authors:** Mamiko Kobayashi, Daisuke Mikami, Junsuke Uwada, Takashi Yazawa, Kazuko Kamiyama, Hideki Kimura, Takanobu Taniguchi, Masayuki Iwano

**Affiliations:** ^1^ Department of Nephrology, Faculty of Medical Sciences, University of Fukui, Fukui, Japan; ^2^ Division of Cellular Signal Transduction, Department of Biochemistry, Asahikawa Medical University, Asahikawa, Japan; ^3^ Department of Clinical Laboratory, University of Fukui Hospital, Fukui, Japan

**Keywords:** GPR41, GPR43, SCFA, cleaved caspase-3, histone deacetylase

## Abstract

Short-chain fatty acids (SCFAs) such as acetate, propionate, and butyrate are generated by microbial fermentation of indigestible fiber by gut flora. SCFAs are ligands of two orphan G protein-coupled receptors, GPR41 and GPR43, that modulate cell proliferation and induce apoptosis. However, it is unclear if SCFAs enhance the effects of chemotherapy in a GPR41- or GPR43-dependent manner. The aim of this study was to investigate whether SCFAs, and particularly propionate, activate GPR41 or GPR43, and thereby enhance the antitumor effects of cisplatin in HepG2 human hepatocellular carcinoma (HCC) cells. The inhibitory effects of propionate and cisplatin on proliferation of HCC cells were determined by MTS assay. Changes in apoptosis rate were analyzed by flow cytometry. The effects of combined propionate and cisplatin on these properties in HCC cells were significantly higher than those of cisplatin alone. With combined treatment, the levels of cleaved caspase-3, active caspase-3 forms, and acetylated histone H3 were enhanced in a GPR41-dependent manner; expression of histone deacetylases (HDAC) 3, 4, 5, 6, 8 proteins was significantly reduced; and induction of TNF-α expression was significantly enhanced. These results suggest that propionate and cisplatin synergistically and significantly induce apoptosis of HepG2 cells by increasing expression of autocrine TNF-α via reduction of HDACs through GPR41 signaling. From clinical and translational perspectives, our data suggest that a combination of propionate with cisplatin may have better therapeutic effects on HCC compared with conventional treatment, and that a selective GPR41 agonist may be a candidate as an adjuvant therapeutic agent for HCC.

## INTRODUCTION

Hepatocellular carcinoma (HCC) is a major malignant cancer worldwide and the third leading cause of cancer death [1]. The mortality rate in most countries is almost equal to the incidence of HCC, indicating the lack of effective treatment. Currently, surgical resection is the most common treatment for HCC. However, the clinical symptoms of HCC are not clear in the early stage, and the disease is often diagnosed at an advanced stage. Patients with inoperable HCC are commonly treated with cisplatin chemotherapy [2], which is effective, but toxic to normal tissue. Therefore, development of a drug with low toxicity that is relatively selective for HCC and with a synergistic effect with established chemotherapeutic drugs may prolong survival of patients with HCC, particularly those in an advanced stage.

Short-chain fatty acids (SCFAs) are a subgroup of fatty acids including six or less carbons. The most important SCFAs are acetate (C2), propionate (C3), and butyrate (C4). In humans, SCFAs are produced exclusively in the gastrointestinal (GI) tract via fermentation of dietary fibers by colonic bacteria. The SCFAs then enter the bloodstream, where butyrate is the main energy source for colonic epithelial cells, propionate is mainly absorbed by the liver, and acetate reaches peripheral tissues [3]. SCFAs are key regulators of immune and metabolic cells, and prevent progression of diseases such as airway disease, colitis, and metabolic disorders [4–6]. SCFAs also affect the cell cycle by inhibiting proliferation and inducing differentiation and apoptosis in human cancer cells [7–9].

SCFAs are thought to exert their effects in two ways: by activating G-protein coupled receptors (GPCRs) and by inhibiting histone deacetylases (HDACs) [10–14]. Recently, two GPCRs, GPR41 and GPR43, have been identified as receptors for SCFAs. In the past decade, SCFAs have been identified as extracellular signaling molecules that mediate regulation of several cellular processes. GPR41 couples to Gi/o signaling pathways and is expressed in gut, adipose tissue, and the peripheral nervous system, while GPR43 couples to Gi/o or Gq signaling pathways [15] and is expressed in adipose tissue, immune cells, and the intestine [16, 17]. Recent data indicate that activation of GPR41 and GPR43 affects tumor growth [8, 9]. Expression of GPR43 is low in most colorectal adenocarcinoma tissues, but high in normal colon tissues [8]. In addition, activation of GPR41 and GPR43 by propionate changes the invasive phenotype of breast cancer cells to a non-invasive phenotype [9].

SCFAs also inhibit cell proliferation by inhibiting HDAC activity and inducing apoptosis [18]. Epigenetic modulation of gene transcription through acetylation and deacetylation of histones plays an important role in the pathogenesis of HCC [19]. Expression of HDAC1-5 and 8 is upregulated in human HCC and enhanced expression of HDACs plays a critical role in malignant growth and immune escape [19, 20]. In contrast to genetic alterations, such epigenetic changes are reversible, and therefore are good therapeutic targets [21]. However, whether HDAC inhibition by SCFAs depends on GPR41 or GPR43 remains unclear. Hence, we hypothesize that SCFAs may contribute to regulation of cancer development by inhibiting HDAC activity through GPR41/43-dependent mechanisms.

The chemotherapeutic agent cisplatin binds to DNA to form intrastrand and interstrand crosslinks between purine bases. A tight chromatin structure prevents cisplatin from approaching DNA, and relaxation of chromatin by HDAC inhibitors increases the accessibility of cisplatin to DNA [22]. However, few studies have examined whether SCFAs enhance the effect of cisplatin chemotherapy by inhibiting HDAC activity by agonism of GPR41 or GPR43. We postulated that a combination of cisplatin with SCFAs would increase the anticancer efficacy of cisplatin. Here, we test this hypothesis by examining augmentation of cisplatin activity by a SCFA, sodium propionate (NaP), in HepG2 cells, and by determining the mechanism underlying the synergistic effect of the combined treatment.

## RESULTS

### Expression of GPR41 and GPR43 in human HCC tissues and cell lines

**Figure 1:**
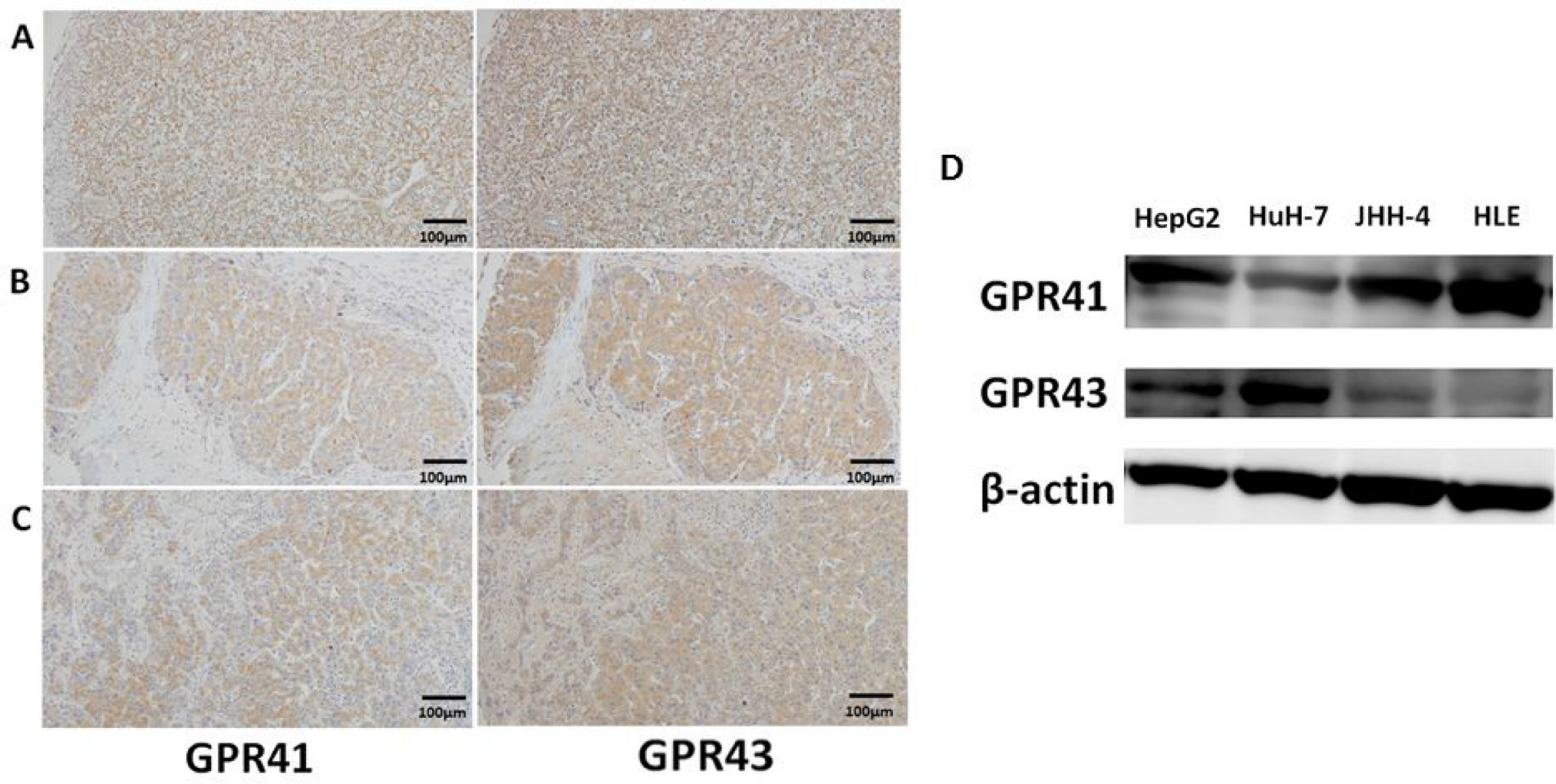
Protein expression of GPR41 and GPR43 in human HCC (**A**–**C**) HCC were immunostained for GPR41 and GPR43. Original magnification ×100. (**D**) GPR41 and GPR43 were analyzed by immunoblotting in whole-cell lysates from confluent HepG2 cells, HuH-7 cells, JHH-4 cells and HLE cells.

GPR41 and GPR43 were expressed at measurable levels in three human HCC tissues with different pathological grades (Figure 1A: grade 1, well differentiated; Figure 1B: grade 2, moderately differentiated; Figure 1C: grade 3, poorly differentiated). Immunoblot analyses showed that GPR41 and GPR43 were present as bands of approximately 39 kDa and 43 kDa, respectively, in all HCC cell lines, including HepG2, HuH-7, JHH-4 and HLE (Figure 1D). Both GPRs seem to be expressed widely in HCC.

### Effects of NaP combined with cisplatin on proliferation and apoptosis of HCC cell lines

**Figure 2:**
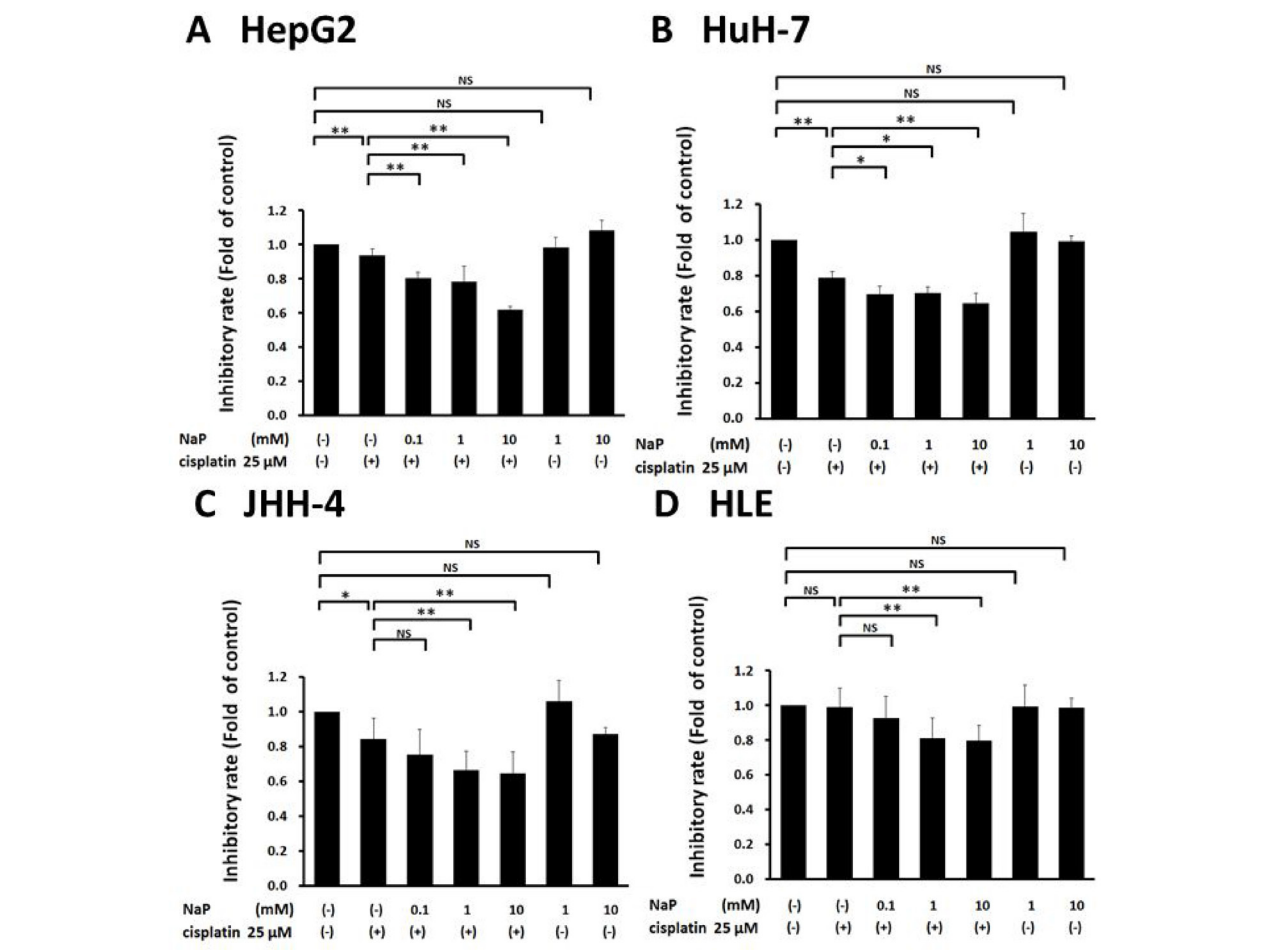
Effects of NaP combined with cisplatin on proliferation rate of HCC cell lines (**A**–**D**) A MTS assay was used to determine the effects of NaP (1, 10 mM) alone, cisplatin (25 µM) alone, or cisplatin (25 µM) + NaP (0.1, 1, 10 mM) on proliferation of HCC cell lines for 24 h. Data are shown as mean ± SD of % apoptosis from three independent experiments. ^*^P < 0.05, ^**^P < 0.01 by one-way ANOVA with a Scheffe *post hoc* test.

**Figure 3:**
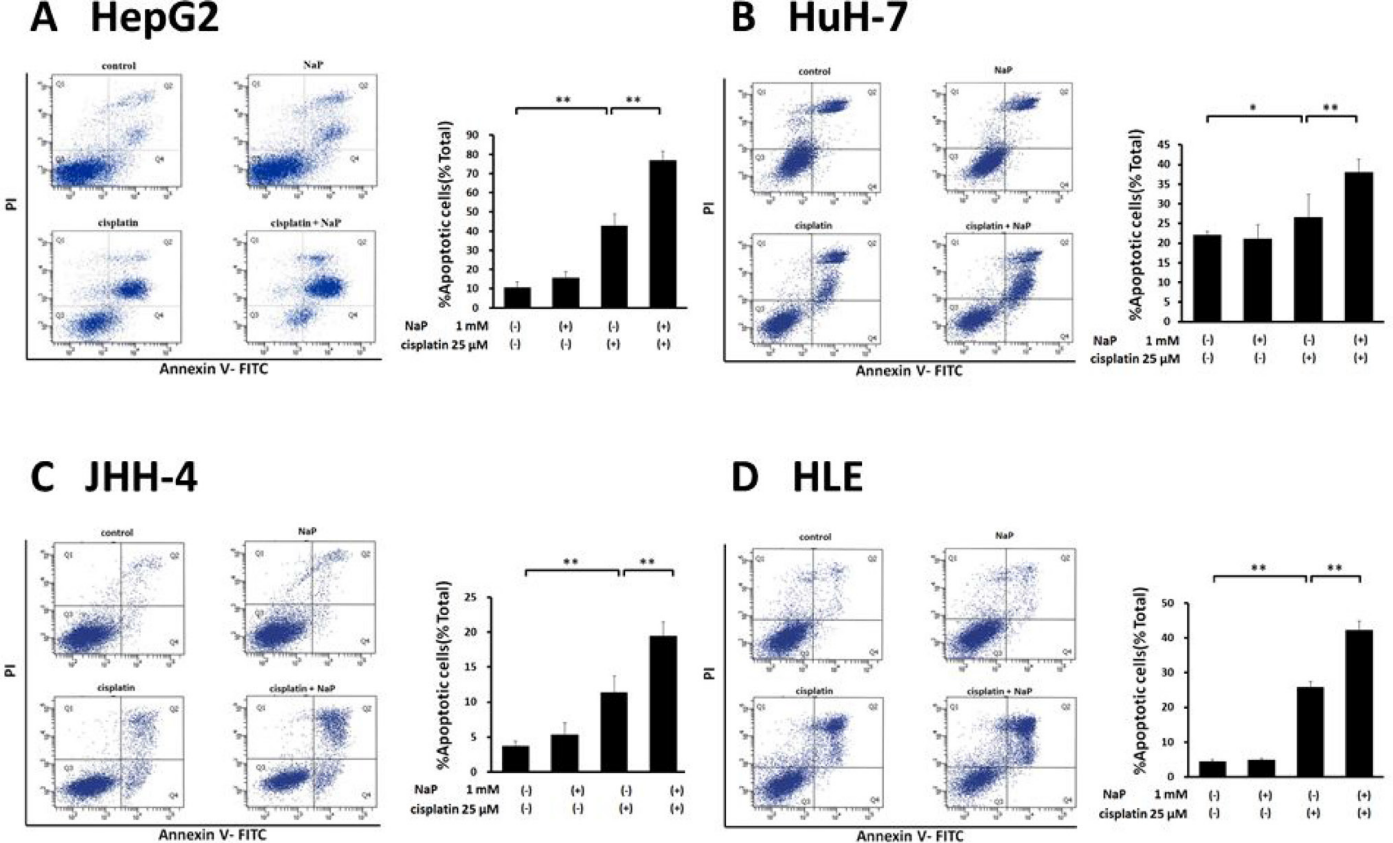
Effects of NaP combined with cisplatin on apoptotic rate of HCC cell lines (**A**–**D**) HCC cells were treated with cisplatin (25 µM), NaP (1 mM), or both agents for 48 h. Cells were then stained with annexin V and PI, followed by cytometry analysis. Data are shown as mean ± SD of % apoptosis from three independent experiments. ^*^P < 0.05, ^**^P < 0.01 by one-way ANOVA with a Scheffe *post hoc* test.

A MTS assay was used to determine the effects of cisplatin (25 µM), NaP (1, 10 mM), and combinations of cisplatin (25 µM) and NaP (0.1, 1, 10 mM) on proliferation of HCC cell lines cells for 24 h. NaP alone did not inhibit proliferation, whereas cisplatin alone significantly inhibited proliferation at 25 µM in HepG2 cells, HuH-7 cells, and JHH-4 cells. The inhibitory effect of NaP + cisplatin on proliferation of HCC cell lines was significantly higher than that of cisplatin alone (*p* < 0.01) with NaP at 0.1 to 10 mM in HepG2 cells and HuH-7 cells (Figure 2A, 2B) and with NaP at 1 to 10 mM in JHH-4 cells and HLE cells (Figure 2C, 2D). In FACS analysis to examine whether NaP (1 mM) enhanced the sensitivity of HCC cell lines to cisplatin, the apoptotic rate at 48 h was significantly higher with NaP + cisplatin than with cisplatin alone in all HCC cell lines (Figure 3A–3D).

### NaP enhances cisplatin-induced apoptosis via GPR41 in HepG2 cells

**Figure 4:**
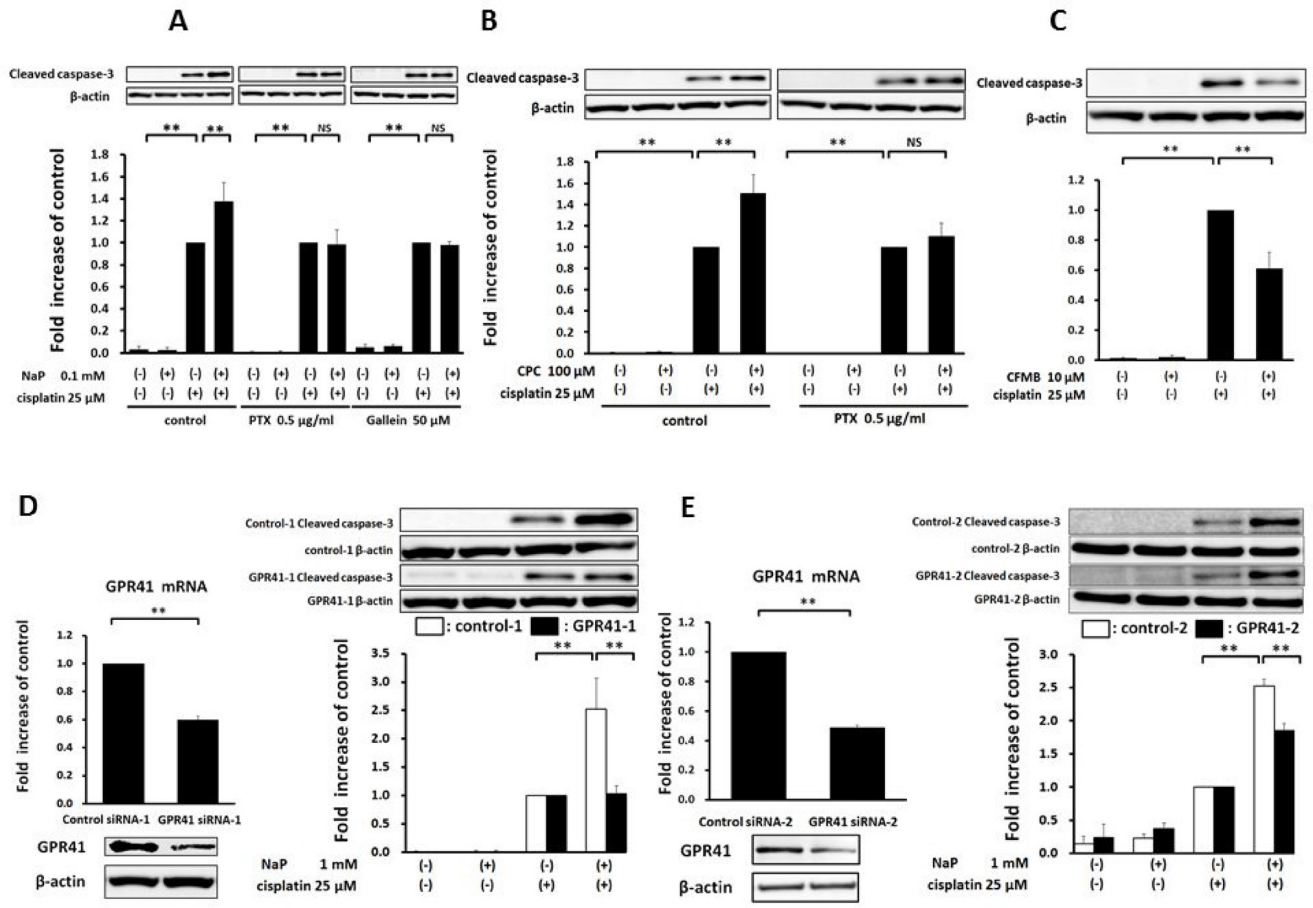
Enhancement of cisplatin-induced apoptosis by NaP in a GPR41-dependent manner in HepG2 cells (**A**) HepG2 cells were treated with cisplatin (25 µM) with or without NaP (0.1 mM) for 24 h. HepG2 cells were treated with vehicle or cisplatin (25 µM) with or without NaP (0.1 mM), and with or without PTX (0.5 mg/ml), or Gallein (50 µM) for 24 h. (**B**) HepG2 cells were treated with vehicle or cisplatin (25 µM) with or without a GPR41 agonist (100 µM CPC) and in the presence of PTX (0.5 mg/ml) for 24 h. (**C**) HepG2 cells were treated with vehicle or cisplatin (25 µM) with or without a GPR43 agonist (10 µM CFMB) for 24 h. (**D**, **E**) Representative mRNA expression (upper panel) and immunoblot of GPR41 protein (lower panel) in siRNA-1-mediated (D) or siRNA-2-mediated (E) GPR41 knockdown cells. HepG2 cells were treated with vehicle or cisplatin (25 µM) with or without NaP (1 mM) in cells treated with control or GPR41 siRNA-1 or siRNA-2. The cleaved caspase-3 level relative to β-actin in cells treated with cisplatin alone was set to 1.0. Data are expressed as the mean ± SD of three separate experiments. ^**^P < 0.01, NS: not significant by one-way ANOVA with a Scheffe *post hoc* test (A–E) and Mann–Whitney test (D, E).

Apoptosis is regulated by highly coordinated processes that involves activation of caspases, which are cysteine proteases [23]. Caspase-3 activation is responsible for DNA fragmentation and myonuclear apoptosis [23, 24]. Therefore, we measured the cleaved, active form of caspase-3 in HepG2 cells by Western blot analysis (Figure 4). NaP + cisplatin significantly increased expression at 0.1 mM NaP (Figure 4A). Next, we examined whether NaP + cisplatin enhanced expression of cleaved caspase-3 via GPR41 or GPR43. Enhancement by NaP was completely blocked by treatment with pertussis toxin (PTX), a Gi/o-type G protein inhibitor [25] (Figure 4A), and was blocked by Gallein, a Gβγ blocker (Figure 4A). We further investigated whether a selective agonist of GPR41 or GPR43 enhanced cisplatin-stimulated expression of cleaved caspase-3. CPC, a GPR41-selective agonist, significantly enhanced cleavage of caspase-3 at 100 µM and the enhancement effect of CPC + cisplatin was blocked by treatment with PTX (Figure 4B). In contrast, CFMB, a GPR43-selective agonist, significantly reduced cleavage at 10 µM (Figure 4C). These data indicate that the enhancement effect of CPC + cisplatin was dependent on a Gi/o signal pathway. GPR41 gene silencing in HepG2 cells using two siRNAs (siRNA-1 and siRNA-2) was performed to clarify whether NaP-mediated enhancement of cisplatin-induced apoptosis was dependent on GPR41. Significant decreases in GPR41 mRNA and protein were found in HepG2 cells treated with siRNAs against GPR41 (Figure 4D, 4E). GPR41 silencing in HepG2 cells significantly blocked NaP-induced enhancement of cisplatin-induced cleaved caspase-3 expression (Figure 4D, 4E). Taken together, these results demonstrate that NaP enhances activation of caspase-3 by cisplatin via a GPR41-mediated pathway.

### NaP enhances cisplatin-induced apoptosis via a TNF-α-induced apoptotic pathway

**Figure 5:**
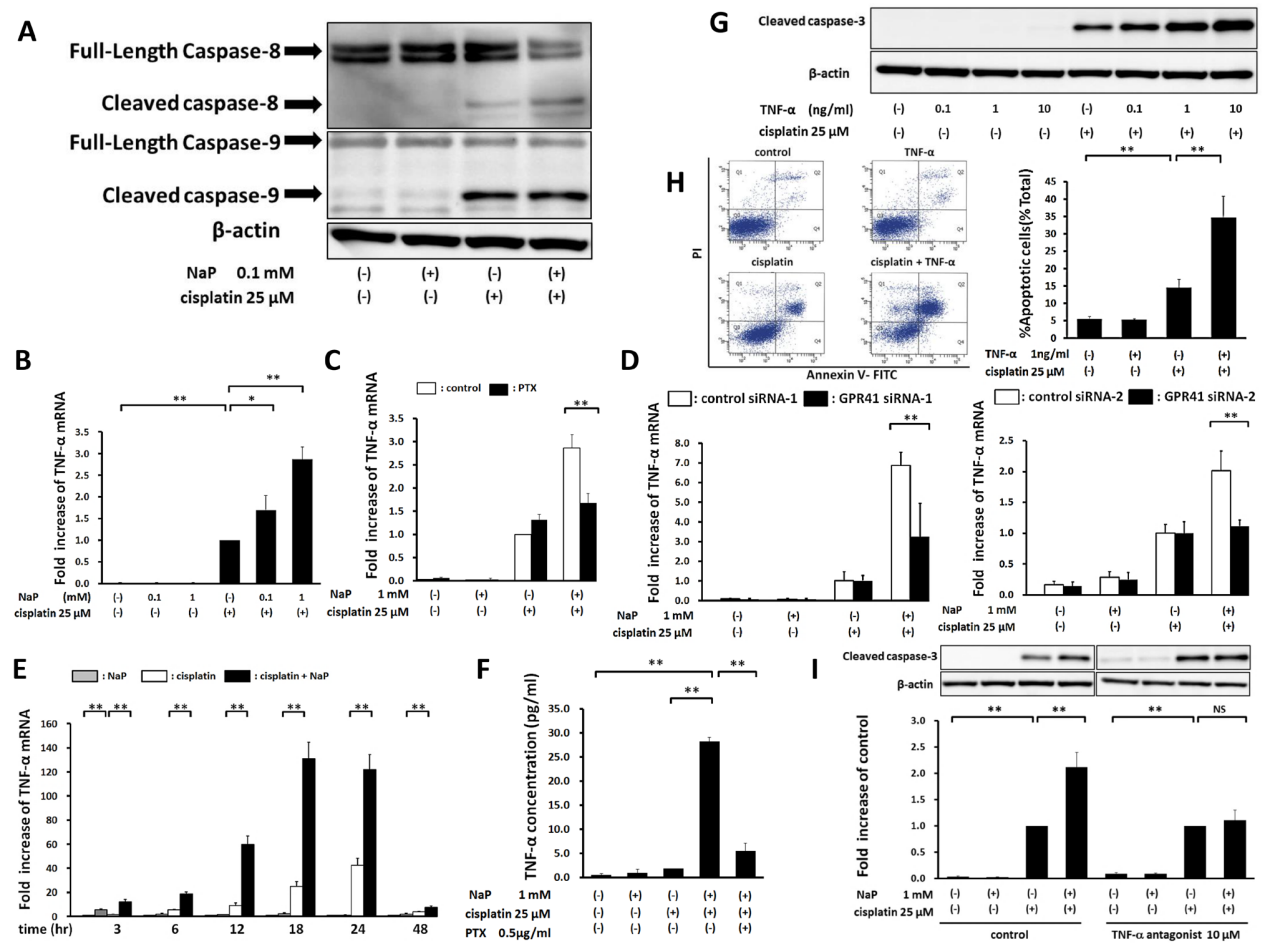
Enhancement of cisplatin-induced apoptosis by NaP through the TNF-α-induced extrinsic apoptotic pathway (**A**) HepG2 cells were treated with vehicle or cisplatin (25 µM) with or without NaP (0.1 mM) for 24 h. Cell lysates were analyzed by western blot for the apoptosis-related proteins, caspase-8 and caspase-9. (**B**) HepG2 cells were treated with vehicle or cisplatin (25 µM) in the presence of the indicated concentration of NaP for 24 h. TNF-α mRNA levels were quantified by TaqMan real-time PCR, with the level in cells treated with cisplatin alone set to 1.0. (**C**) HepG2 cells were treated with vehicle or cisplatin (25 µM) with or without NaP (1 mM) and with or without PTX (0.5 mg/ml) for 24 h. TNF-α mRNA levels were quantified by TaqMan real-time PCR, with the level in cells treated with cisplatin alone set to 1.0. (**D**) HepG2 cells were treated with vehicle or cisplatin (25 µM) with or without NaP (1 mM) for 24 h in cells treated with control or GPR41 siRNA-1/2. TNF-α mRNA levels were quantified by TaqMan real-time PCR, with the level in cells treated with cisplatin alone set to 1.0. (**E**) HepG2 cells were treated with vehicle or cisplatin (25 µM) with or without NaP (1 mM) for 3, 6, 12, 18, 24, and 48 h. TNF-α mRNA levels were quantified by TaqMan real-time PCR, with the level in cells treated with vehicle set to 1.0. (**F**) HepG2 cells were treated with vehicle or cisplatin (25 µM) with or without NaP (1 mM) or PTX (0.5 mg/ml) for 24 h. TNF-α in supernatants was quantified by ELISA. (**G**) HepG2 cells were treated with vehicle or human recombinant TNF-α (0.1, 1, 10 ng/ml) or cisplatin (25 µM) in the presence of the indicated concentration of human recombinant TNF-α for 24 h. (**H**) HepG2 cells were treated with cisplatin (25 µM), TNF-α (1 ng/ml), or both agents for 24 h. Cells were then stained with annexin V and PI, followed by cytometry analysis. (**I**) HepG2 cells were treated with vehicle or cisplatin (25 µM) with or without NaP (1 mM) and with or without a TNF-α antagonist (10 µM) for 24 h. Cell lysates were analyzed by western blot for cleaved caspase-3, with the cleaved caspase-3 level relative to β-actin in cells treated with cisplatin alone set to 1.0. Data are expressed as the mean ± SD of three separate experiments. ^*^P < 0.05, ^**^P < 0.01, NS: not significant by one-way ANOVA with a Scheffe *post hoc* test (B–F, H, I).

To investigate the molecular mechanism of NaP enhancement of cisplatin-induced apoptosis in HepG2 cells, immunoblotting was performed for caspase family members that might function during apoptosis (Figure 5A). Cisplatin treatment induced cleavage of caspase-8 and -9, and the protein level of activated caspase-8 with NaP + cisplatin was significantly higher than that with cisplatin alone. NaP at 0.1 and 1 mM significantly induced expression of mRNA for TNF-α, a pro-apoptotic cytokine, in a dose-dependent manner in HepG2 cells treated with cisplatin (Figure 5B). Enhancement of cisplatin-induced TNF-α expression by NaP was blocked by treatment with PTX (Figure 5C). GPR41 silencing in HepG2 cells significantly reduced NaP-induced enhancement of cisplatin-induced expression of mRNA for TNF-α (Figure 5D). Expression of mRNA for TNF-α with NaP + cisplatin was significantly higher than that with cisplatin alone from 3 h to 48 h (Figure 5E). NaP at 1 mM significantly increased secretion of TNF-α, and this was significantly blocked by PTX (Figure 5F). Recombinant TNF-α significantly enhanced cisplatin-induced production of cleaved caspase-3 in a dose-dependent manner (Figure 5G) and the apoptotic rate at 24 h was significantly higher with TNF-α + cisplatin than with cisplatin alone in FACS analysis (Figure 5H). Enhancement of cleaved caspase-3 by NaP + cisplatin was significantly blocked by treatment with a TNF-α antagonist (Figure 5I). Taken together, these results show that NaP enhances cisplatin-induced apoptosis through a TNF-α-induced extrinsic apoptotic pathway via GPR41.

### NaP enhances cisplatin-induced acetylation of histone H3 via GPR41 in HepG2 cells

**Figure 6:**
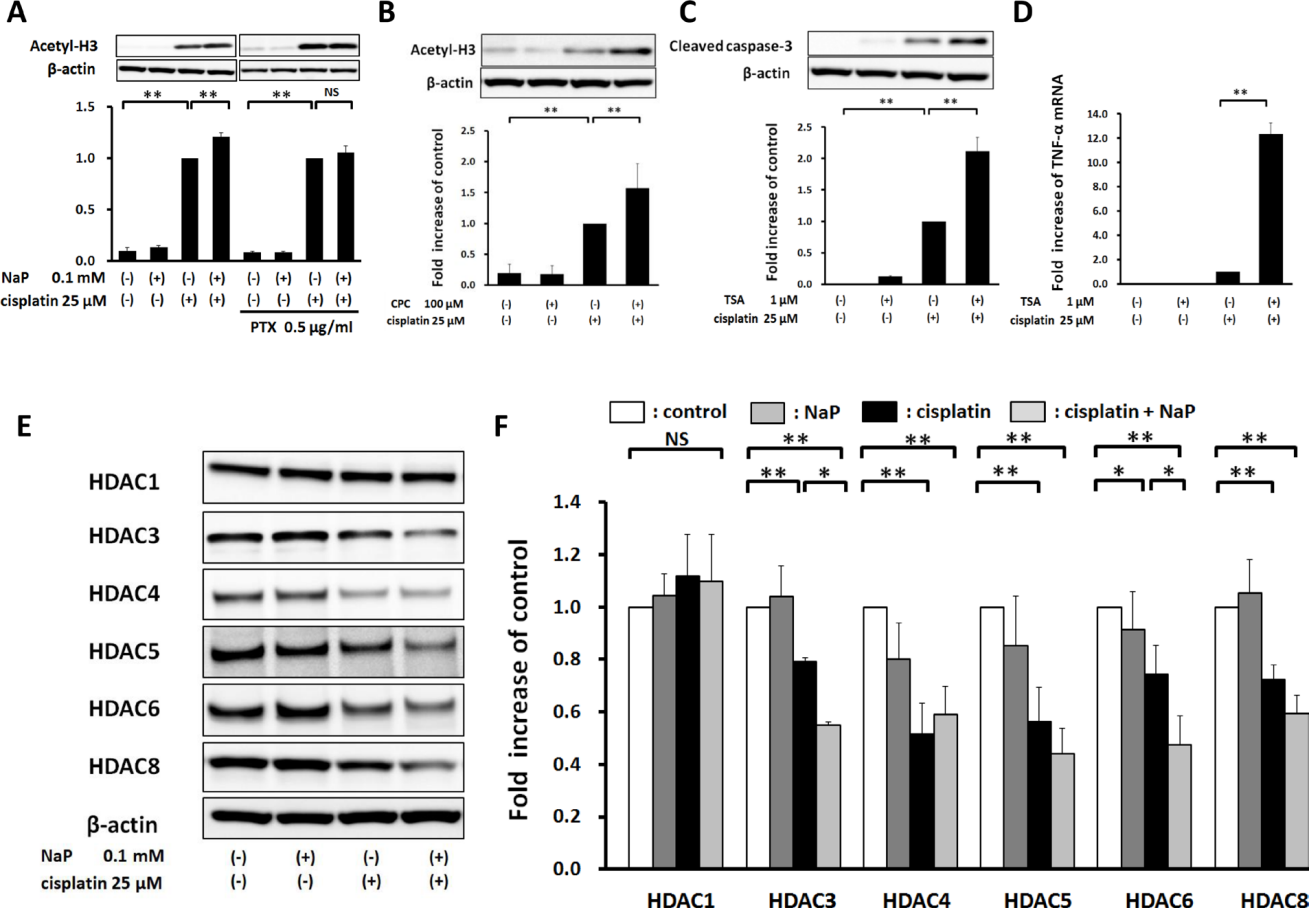
Elevation of cisplatin-induced acetylation of histone H3 by NaP in a GPR41-dependent manner in HepG2 cells (**A**) HepG2 cells were treated with vehicle or cisplatin (25 µM) with or without NaP (0.1 mM) and with or without PTX (0.5 mg/ml) for 24 h. Cell lysates were analyzed by western blot for acetylation of histone H3. (**B**) HepG2 cells were treated with vehicle or cisplatin (25 µM) with or without a GPR41 agonist (100 µM CPC) for 24 h. Cell lysates were analyzed by western blot for acetylation of histone H3. (**C**, **D**) HepG2 cells were treated with vehicle or cisplatin (25 µM) with or without TSA (1 µM) for 24 h. Cell lysates were analyzed by western blot for an apoptosis-related protein, cleaved caspase-3. TNF-α mRNA levels were quantified by TaqMan real-time PCR, with the level in cells treated with cisplatin alone set to 1.0. (**E**, **F**) HepG2 cells were treated with vehicle or cisplatin (25 µM) with or without NaP (0.1 mM) for 24 h. Cell lysates were analyzed by western blot for HDAC1, 3, 4, 5, 6 and 8. The levels of acetyl-H3 and cleaved caspase-3 (all relative to β-actin) were set to 1.0 in cells treated with cisplatin alone. The levels of HDACs (all relative to β-actin) were set to 1.0 in cells treated with vehicle. Data are expressed as the mean ± SD of three separate experiments. ^*^P < 0.05, ^**^P < 0.01, NS not significant by one-way ANOVA with a Scheffe *post hoc* test (A–D, F).

Next, we investigated whether NaP enhanced cisplatin-induced apoptosis through a HDAC inhibitory pathway via GPR41. NaP significantly enhanced cisplatin-induced acetylation of histone H3 at 0.1 mM (Figure 6A), and this effect was completely blocked by PTX (Figure 6A). CPC also enhanced acetylation of histone H3 (Figure 6B). TSA, a pan-HDAC inhibitor, significantly enhanced cisplatin-induced expression of cleaved caspase-3 at 1 µM (Figure 6C), and significantly enhanced cisplatin-induced TNF-α expression (Figure 6D). To investigate whether treatment with cisplatin or NaP + cisplatin decreased expression of HDACs, which lead to acetylation of histones, Western blot analysis was used to examine expression of HDACs in HepG2 cells. Cisplatin alone decreased the levels of HDAC3, 4, 5, 6, and 8, and NaP + cisplatin further decreased HDAC3 and 6 (Figure 6E, 6F). This indicates that enhancement of acetylation of histone H3 by NaP occurs partially through a HDAC3, 6-dependent pathway. Taken together, these results show that NaP enhances acetylation of histone H3 through reducing the expression of several HDACs in combination treatment with cisplatin in a GPR41-dependent manner.

### Efficacy of NaP combined with cisplatin in hepatocellular carcinoma HepG2 xenografts

**Figure 7:**
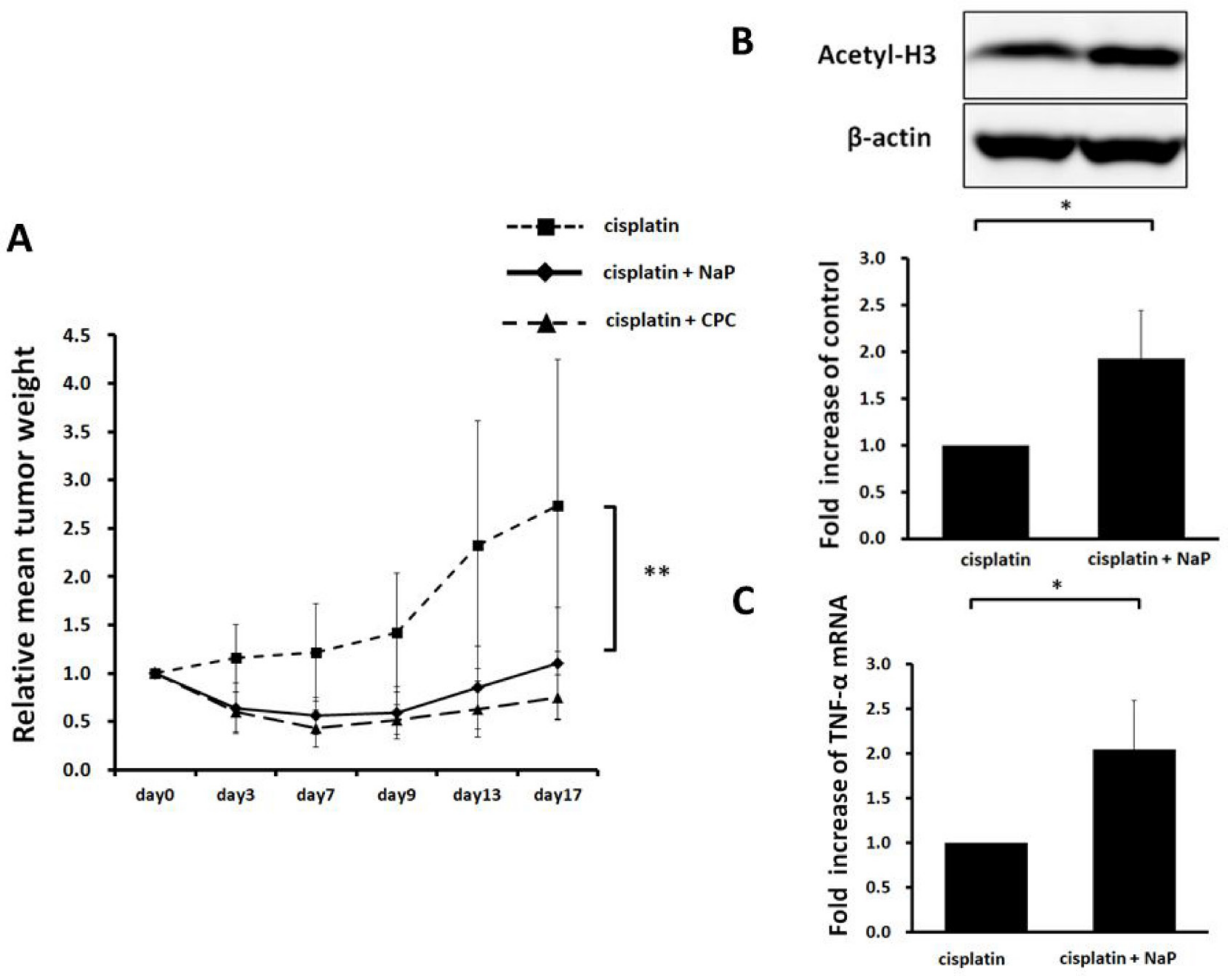
Therapeutic efficacy of NaP + cisplatin in HCC HepG2 xenografts Six-week-old female SHO nude mice were subcutaneously injected with 1.0 x 10^6^ cells. When the tumor size reached 256-500 mm^3^, mice were treated by intraperitoneal injection of 8 mg/kg cisplatin or 8 mg/kg cisplatin + 250 mg/kg NaP or 8 mg/kg cisplatin + 50 mg/kg CPC dissolved in saline. (**A**) Xenografts were measured as described in the Materials and Methods. Results are expressed as the mean ± SD (*n* = 5). (**B**) Acetylation of histone H3 in HepG2 tumors was evaluated by Western blotting at 4 days after treatment with 8 mg/kg cisplatin or 8 mg/kg cisplatin + 250 mg/kg NaP (*n* = 5). (**C**) Expression of mRNA for TNF-α in HepG2 tumors was evaluated by Western blotting at 4 days after treatment with 8 mg/kg cisplatin or 8 mg/kg cisplatin + 250 mg/kg NaP (*n* = 5). ^*^P < 0.05, ^**^P < 0.01, NS: not significant by Student *t*-test.

A HepG2 xenograft model was used for evaluation of therapeutic efficacy *in vivo*. NaP (250 mg/kg) + cisplatin or CPC (50 mg/kg) + cisplatin significantly suppressed growth of HepG2 xenografts (*p* < 0.01) (Figure 7A). NaP + cisplatin significantly enhanced acetylation of histone H3 (Figure 7B) and expression of TNF-α mRNA and in the xenografts at day 4 (Figure 7C).

## DISCUSSION

In this study, we showed that GPR41 and GPR43 are moderately expressed in human HCC, and we found that a SCFA, propionate, enhanced the chemosensitivity of HepG2 cells to cisplatin by inducing TNF-α expression via GPR41 activation involving Gi/o and particularly Gβ/γ. To the best of our knowledge, this is the first report to describe enhancement of the effect of cisplatin chemotherapy by propionate in HCC cell.

SCFAs are produced by microbial fermentation of indigestible fiber by anaerobic gut flora and are important energy sources for the host. GPR41 and GPR43 are recently identified GPCRs for SCFAs [15], and there is growing evidence that SCFAs have therapeutic potential for protection against various inflammatory disease via GPR41 or GPR43 [26–28]. We also recently reported that SCFAs, especially propionate, attenuate TNF-α-stimulated monocyte chemotactic protein-1 (MCP-1) expression by inhibiting phosphorylation of p38 and JNK in human renal cortical epithelial cells [29], and we showed that this inhibitory effect occurred in a GPR41/GPR43-dependent manner [29]. Furthermore, recent data suggest that GPR41 and GPR43 play a role in tumor suppression [7, 8]. Propionate, the most potent agonist of GPR41 and GPR43, reduces proliferation and promotes apoptosis in colon cancer [8] and leukemia [7], but it is unclear if propionate enhances the effect of chemotherapy on cancer cells. In this study, we showed that a combination of cisplatin and propionate (NaP) significantly inhibited growth and increased apoptosis in HepG2 cells.

GPR41 and GPR43 are SCFA receptors that couple with Gi/o and with Gi/o or Gq, respectively [15]. In the intestine, GPR43 promotes glucagon-like peptide-1 (GLP-1) secretion in L cells through a Gq-coupled pathway [30], and a recent study showed that acetate activates p38 phosphorylation in human monocytes, probably via Gq, but not Gi/o, signaling [31]. In the current study, NaP and CPC, a selective agonist of GPR41, significantly enhanced the cisplatin-induced activation of caspases. In contrast, CFMB, a selective agonist of GPR43, reduced the activation, and this reduction was significantly blocked in a Gq inhibitor (YM254890)-sensitive manner (data not shown). Based on these findings, enhancement of apoptosis by NaP is dependent on GPR41 activation involving Gi/o. On the other hand, selective stimulation of GPR43 may reduce cisplatin-induced apoptosis involving Gq.

A key finding in the current study is that NaP enhanced cisplatin-induced apoptosis by inhibiting HDAC, an epigenetic modifier, via GPCR activation. A recent study showed that SCFAs inhibited production of pro-inflammatory cytokines, oxidative cellular stress, and cell infiltration by inhibiting HDAC, independent of GPR41 and GPR43, in mice with ischemic nephropathy [32], and in neutrophils, SCFAs inhibit HDAC independently of GPR41 and GPR43 [33]. On the other hand, in colon tissue, HDAC inhibition by SCFAs is partially dependent on GPR43 [34]. The current study showed that NaP inhibited HDACs via GPR41 in HepG2 cells. Thus, effects of GPR41 and GPR43 on HDAC inhibition may be cell-specific.

Another major finding of the study was the enhancement of cisplatin-induced production of TNF-α by NaP via HDAC inhibition in HepG2 cells. Cisplatin is actively transported into cells, and this is followed by activation of several stress signaling cascades such as the p53 signaling pathway, mitogen-activated protein kinase (MAPK) signaling pathway, and both intrinsic and extrinsic apoptosis pathways [35, 36] The extrinsic pathway for apoptosis is induced through activation of death receptors including CD95 receptor (Fas) and tumor necrosis factor receptor 1 (TNFR1), leading to activation of caspase-8 [37]. In the current study, we found that a combination of NaP and cisplatin enhanced cleavage of caspase-3 and -8 significantly compared with cisplatin alone. In addition, expression of TNF-α mRNA and protein were enhanced significantly by NaP and cisplatin compared with cisplatin alone, and this enhancement was blocked by treatment with PTX. Recombinant TNF-α and cisplatin enhanced cleavage of caspase-3 in a dose-dependent manner, and further enhancement of NaP with cisplatin was completely blocked by a TNF-α antagonist.

Modulation of autocrine TNF-α can sensitize cancer cells to cisplatin [38]. We found that cisplatin alone decreased the levels of HDAC3, 4, 5, 6, and 8 and that addition of NaP to cisplatin further decreased HDAC3 and 6. A previous study showed that suppression of HDAC3 or 5 induced apoptosis in HCC cell lines [39, 40]. Similarly, knockdown of HDAC4 enhanced radiation-induced cell death, that of HDAC6 reduced the migration and invasion activities of all HCC cell lines [41], and that of HDAC8 repressed tumor cell growth and induced apoptosis [42]. A recent study showed that NaP at 0.1 mM reduced expression of mRNA of HDAC6 in mouse colonic regulatory T cells [34]. In the current study, we found that NaP at 0.1 mM did not reduce HDAC6 mRNA expression significantly (data not shown). Therefore, the effects of NaP on HDAC expression may be cell and/or species-dependent. We also found that a combination of cisplatin and TSA, a potent specific pan-HDAC inhibitor [43], including HDAC1, 2, 3, 4, 6 and 10, increased TNF-α expression significantly. These findings suggest that a combination of cisplatin and NaP enhances apoptosis by increasing expression of autocrine TNF-α via reduction of HDACs in HepG2 cells. However, the molecular mechanism downstream of GPR41 to HDACs remains unclear. Finally, in an *in vivo* study in xenograft tumors, we found combination treatment of cisplatin and NaP significantly suppressed growth of HepG2 cells and enhanced acetylation of histone H3, compared with cisplatin alone.

**Figure 8:**
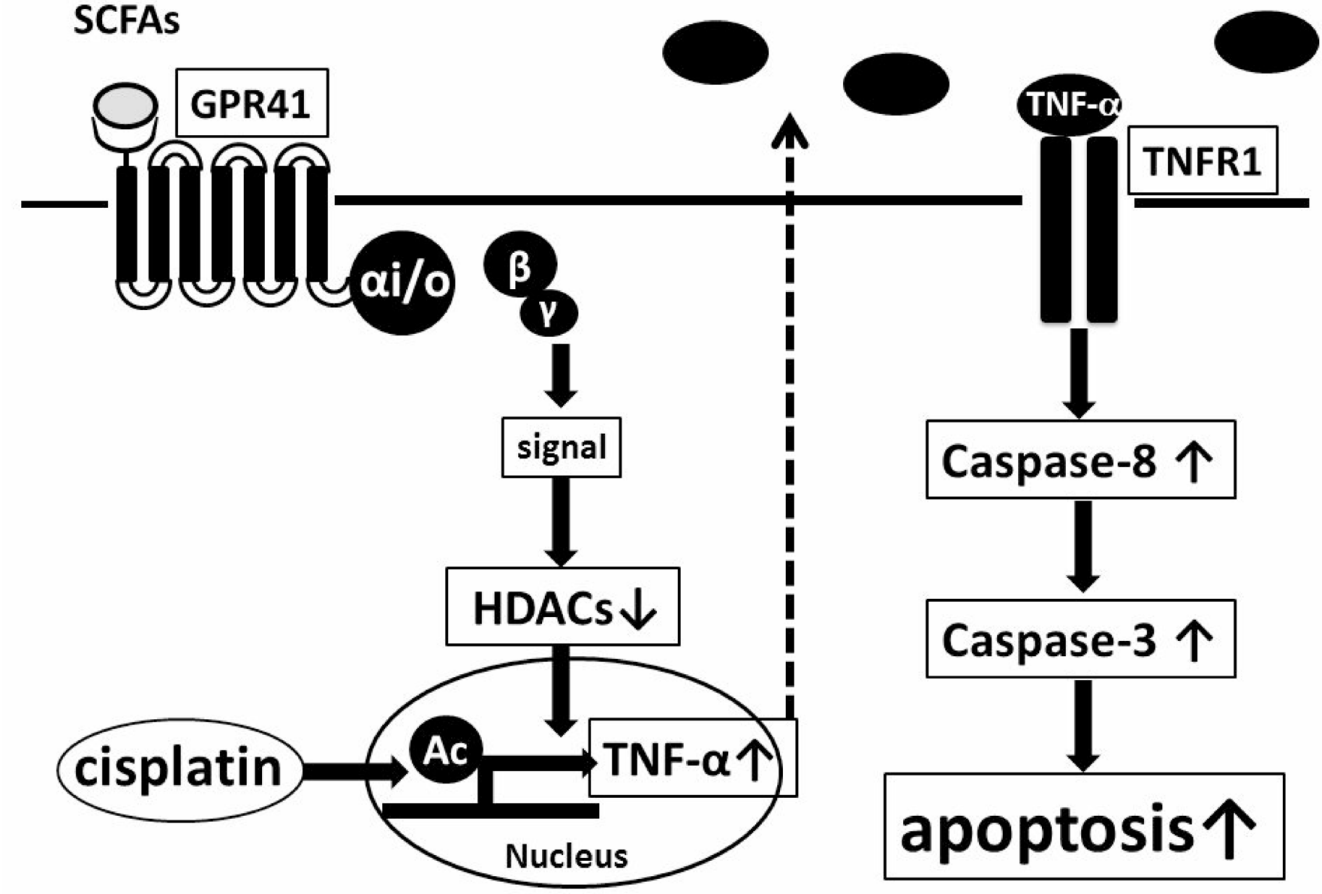
Model of enhancement of cisplatin-induced apoptosis in HepG2 cells mediated by GPR41, a SCFA receptor Propionate, a gut-microbiota product, enhances proliferation of cisplatin-induced cell apoptosis of HepG2 cells by increasing expression of autocrine TNF-α via reduction of HDACs in a GPR41-dependent manner.

In conclusion, the synergism of propionate and cisplatin significantly enhances inhibition of proliferation and induction of apoptosis of HepG2 cells by increasing expression of autocrine TNF-α via reduction of HDACs in a GPR41-dependent manner (summarized in Figure 8). Our data suggest that combined treatment of propionate with cisplatin may exert better therapeutic effects on HCC compared with conventional treatment. Finally, a selective agonist of GPR41 might serve as a novel adjuvant therapeutic agent for treatment of HCC.

## MATERIALS AND METHODS

### Materials

(S)-2-(4-chlorophenyl)-3,3-dimethyl-N-(5-phenylthiazol-2-yl) butanamide (CFMB) and TNF-α antagonist R-7050 [44] (Calbiochem, Darmstadt, Germany); sodium propionate (NaP) (Sigma-Aldrich, St. Louis, MO, USA); pertussis toxin (PTX) (Wako, Osaka, Japan); gallein, cyclopropanecarboxylic acid (CPC) and trichostatin A (TSA) (Tokyo Chemical Industry, Tokyo, Japan); human recombinant TNF-α (PeproTech, Rocky Hill, NJ, USA); polyclonal rabbit antibodies against human β-actin (Abcam), acetyl-histone H3 (Lys9/Lys14), cleaved caspase-3 (Asp175), cleaved caspase-9 (all Cell Signaling Technology, Boston, MA USA), GPR41 (Abcam), and GPR43 (Bioss, Boston, MA, USA); monoclonal rabbit antibodies against HDAC1, HDAC4, HDAC5 (Cell Signaling Technology) and HDAC8 (Abcam); monoclonal mouse antibodies against HDAC3 and caspase-8 (Cell Signaling Technology); and horseradish peroxidase-conjugated anti-mouse, anti-goat and anti-rabbit immunoglobulins (Dako, Glostrup, Denmark) were used in the study.

### Cell cultures

HepG2 cells, HuH-7 cells, JHH-4 cells and HLE cells, which are all human hepatoma cell lines, were obtained from the Japanese Collection of Research Bioresources Cell Bank. HepG2, HuH-7 and HLE cells were grown in Dulbecco’s modified Eagle’s medium (DMEM) with 10% fetal calf serum supplemented with 10% FBS and 1% penicillin/streptomycin, and JHH-4 cells were grown in Eagle’s minimal essential medium with 10% fetal calf serum supplemented with 10% FBS and 1% penicillin/streptomycin at 37° C in a humidified atmosphere containing 5% CO_2_ and 18% O_2_. When cells reached a logarithmic phase of growth, they were digested with trypsin and seeded in different culture plates based on experimental needs.

### Cell proliferation assays

Cell proliferation was evaluated by MTS assay (Promega, Madison, WI, USA). HCC cells were seeded in 96-well plates at 5 × 10^3^ cells per well, and treated with cisplatin (25 µM) alone, NaP (1, 10 mM) alone, or a combination of cisplatin (25 µM) and NaP (0.1, 1, 10 mM) at 37° C under 5% CO_2_ for 24 h. At the end of the 24-h period, 20 µl MTS reagent supplied in the CellTiter 96 Aqueous Assay was added to each well, and the plates were incubated for a further 4 h at 37° C under 5% CO_2_. The absorbance of the product was measured at 490 nm using a microplate reader, with a reference wavelength of 650 nm.

### TaqMan real-time PCR assay

TaqMan real-time RT-PCR was performed as previously reported [45]. Specific primers and TaqMan MGB probes (Applied Biosystems) were used to detect human TNF-α (assay ID: Hs00174128_m1). A TaqMan human β-actin MGB control reagent kit (Applied Biosystems) was used to detect human β-actin. Expression of TNF-α mRNA was normalized to that of β-actin mRNA.

### Knockdown of GPR41 in HepG2 cells

Gene silencing of GPR41 in HepG2 cells was performed as described previously [45]. Small interfering RNA (siRNA) against GPR41 (siGENOME Human FFAR3: GPR41 siRNA-1; and ON-TARGET plus Human FFAR3: GPR41 siRNA-2), and control siRNA (siGENOME Non-siRNA: control siRNA-1; and ON-TARGET plus Non-targeting: control siRNA-2) were purchased from GE Healthcare Dharmacon (Lafayette, CO, USA). HepG2 cells (70% confluence) were transfected with control siRNA or siRNA against GPR41 at a final concentration of 50 nmol/L, using a transfection reagent (DharmaFECT; Dharmacon). After a 72-h incubation, HepG2 cells were refreshed with DMEM and then treated for a further 24 h with cisplatin with or without NaP.

### Determination of human TNF-α concentrations

The TNF-α level in each cell culture supernatant was measured by immunoassay using an ELISA kit (Quantikine Human TNF-α; R&D Systems, Minneapolis, MN, USA).

### Immunoblot analysis

HepG2 cells were lysed in RIPA buffer with phosphatase inhibitors (Sigma-Aldrich). Lysates (5 µg protein) were analyzed by immunoblot analysis, first using antibodies targeting GPR41 (1:1000), GPR43 (1:1000), β-actin (1:1000), cleaved caspase-3 (1:1000), caspase-8 (1:1000), caspase-9 (1:1000), acetyl-H3 (1:1000), HDAC1 (1:1000), HDAC3 (1:1000), HDAC4 (1:1000), HDCA5 (1:1000), HDAC6 (1:1000), and HDAC8 (1:5000) for 24 h, and then with appropriate horseradish peroxidase-conjugated secondary antibodies (1:1000) at room temperature for 1 h. Immunoreactive bands were visualized as previously reported [45].

### Immunohistochemistry

HCC samples were purchased from Biomax. Immunoperoxidase staining was performed with rabbit polyclonal antibodies against GPR41 (1:200) and GPR43 (1:200) as primary antibodies to determine the localization of GPR41 and GPR43. Positive staining was detected using an Envision kit/HRP (DAB) (Dako).

### Cell apoptosis quantification

An annexin V kit (MBL, Nagoya, Japan) was used to evaluate apoptosis in HCC cells. Cells were resuspended in 85 µl of binding buffer. The suspension was incubated with 10 µl of Annexin V-FITC and 5 µl of propidium iodide (PI) for 15 min at room temperature in the dark. Then, 400 µl of binding buffer was added to each tube and the percentage of apoptotic cells was quantified by flow cytometry using a BD FACSCanto™ II Flow Cytometer.

### *In vivo* xenograft study

Animals were housed in appropriate pathogen-free conditions. All experimental procedures conformed to the Regulations for Animal Research at the University of Fukui, and were reviewed by the Animal Research Committee of the University of Fukui. Mice were housed under standard conditions (12 h light/12 h dark). Six-week-old male SHO nude mice (Charles River, Japan) were subcutaneously injected with 1.0 × 10^6^ HepG2 cells. When the tumor size reached 256–500 mm^3^, mice were randomly allocated to two groups (*n* = 5 per group) and treated by intraperitoneal injection of 8 mg/kg cisplatin or 8 mg/kg cisplatin plus 250 mg/kg NaP, both dissolved in saline. The tumor size was measured every 3 days with calipers. The tumor weight was estimated as (length × width^2^)/2. To standardize the variability in tumor weights among the treated groups, the relative tumor weights (RTWs) at different times were obtained using the formula TWi/TWo, where TWi is the mean tumor weight of a group on day n and TWo is the mean tumor weight on day 0. The relative mean body weight was calculated using the formula BW17/BWo, where BW17 is the mean body weight of a group on day 17 and BWo is the mean body weight on day 0. In an independent experiment, HepG2 tumor homogenates were prepared by collecting tumors 4 day after IP treatment with 8 mg/kg cisplatin or 8 mg/kg cisplatin plus 250 mg/kg NaP or 8 mg/kg cisplatin plus 50 mg/kg CPC (*n* = 5 per group). Tumors were frozen with liquid nitrogen and stored at –80° C prior to analysis.

### Statistical analysis

Data are expressed as the mean ± the standard deviation (SD). The significance of differences between two groups was evaluated by Man–Whitney test or Student *t*-test, and that among more than two groups was assessed using analysis of variance (ANOVA) with a Scheffe *post hoc* test. Results were considered to be significant at *P* < 0.05.

## Abbreviations

CFMB: (S)-2-(4-chlorophenyl)-3,3-dimethyl-N-(5-phenylthiazol-2-yl) butanamide
CPC: cyclopropanecarboxylic acid
DMEM: Dulbecco’s modified Eagle’s medium
GLP-1: glucagon-like peptide-1
GPCR: G-protein coupled receptor
HCC: Hepatocellular carcinoma
HDAC: histone deacetylase
MAPK: mitogen-activated protein kinase
MCP-1: monocyte chemotactic protein-1
NaP: sodium propionate
PTX: pertussis toxin
RTWs: relative tumor weights
SCFA: Short-chain fatty acid
siRNA: small interfering RNA
TNFR1: tumor necrosis factor receptor 1
TSA: trichostatin A
